# Endovascular Revascularization with Stent Implantation in Patients with Acute Mesenteric Ischemia due to Acute Arterial Thrombosis: Clinical Outcome and Predictive Factors

**DOI:** 10.1007/s00270-021-02824-2

**Published:** 2021-04-06

**Authors:** Federico Pedersoli, Kai Schönau, Maximilian Schulze-Hagen, Sebastian Keil, Peter Isfort, Alexander Gombert, Patrick Hamid Alizai, Christiane K. Kuhl, Philipp Bruners, Markus Zimmermann

**Affiliations:** 1 Department of Diagnostic and Interventional Radiology, University Hospital RWTH Aachen, Pauwelsstraße 30, 52074 Aachen, Germany; 2 Department of Vascular Surgery, University Hospital RWTH Aachen, Pauwelsstraße 30, 52074 Aachen, Germany; 3 Department of General, Visceral and Transplant Surgery, University Hospital RWTH Aachen, Pauwelsstraße 30, 52074 Aachen, Germany

**Keywords:** Mesenteric ischemia, Celiac artery, Superior mesenteric artery, Endovascular procedures, Stents

## Abstract

**Purpose:**

To determine 30-day-mortality rates and identify predictors for survival in patients undergoing endovascular revascularization for acute mesenteric ischemia (AMI) due to occlusion of the celiac (CA) or superior mesenteric artery (SMA) from arterial thrombosis in the setting of atherosclerosis at the vessel origin.

**Materials and Methods:**

A retrospective analysis on patients who underwent acute endovascular revascularization to treat AMI caused by thrombotic occlusion of the CA and/or SMA between January 2011 and December 2019 was conducted. 30-day-mortality rates were calculated. Univariate binomial logistic regression analyses (*p* < 0.05) were performed to assess whether the following factors were associated with 30-day mortality: sex, age, history of smoking, history of abdominal angina, signs of bowel necrosis on pre-interventional CT, one- vs. two-vessel disease, patency of the inferior mesenteric artery, outpatient or inpatient occurrence of ischemia, onset of AMI during ITU stay, elevated pre-interventional serum lactate levels, total leukocyte count, platelet/lymphocyte ratio and neutrophil/lymphocyte ratio.

**Results:**

40 patients were included in this analysis. 30-day-mortality rate was 25/40 (62.5%). Median overall survival of patients who survived the first 30 days was 36 ± 18 months. None of the analyzed factors was statistically significantly associated with 30-day mortality.

**Conclusion:**

Although mortality of patients with AMI due to acute arterial thrombosis remains high, almost 40% of patient who underwent emergent endovascular revascularization survived longer than one month. Since no predictors for the outcome in these patients were identified, all patients with AMI should be offered an immediate revascularization effort.

## Introduction

Acute mesenteric ischemia (AMI) is a potentially life-threatening condition caused by insufficient blood flow to the bowel and visceral organs due to either arterial embolism, arterial thrombosis, mesenteric venous thrombosis or non-occlusive mesenteric ischemia (NOMI). AMI carries a poor prognosis [1]: the cumulative in-hospital mortality rate has been reported to be as high as 70% [2]. Arterial thrombosis accounts for 25–30% of all cases of acute mesenteric ischemia [3] and usually occurs as a result of an acute occlusion near the origins of the superior mesenteric artery (SMA) or the celiac artery (CA), often in the setting of chronic atherosclerotic disease.

Bowels can tolerate a reduction of mesenteric blood flow up to 75% for 12 h [4], because of many collateral vessels between the three main arteries responsible for bowel perfusion: the CA, the SMA and inferior mesenteric artery (IMA) [5]. However, a complete occlusion of one of the mesenteric vessels can lead to irreversible damage to the bowel wall within 6 h [6, 7]. Consequently, emergency restitution of visceral blood flow is of paramount importance to avoid wide-spread irreversible intestinal necrosis [6].

Several factors have been advocated to be possible predictive factors for the outcome of patients with AMI: serum lactate levels [8, 9], leucocytosis [8], ratio between platelets and lymphocytes (PLR) [10] or between neutrophils and lymphocytes (NLR), signs of mesenteric ischemia on computer tomography (CT) [9], occurrence of AMI during stay in an intensive therapy unit (ITU) [11] and advanced age [12]. Nevertheless, the majority of studies on the outcome for patients with AMI include patients with different causes of AMI, i.e., embolic, thrombotic, etc., and therefore there is a lack of data on specific predictive factors for AMI resulting from arterial thrombosis.

The purpose of this study was to evaluate the outcome of patients undergoing endovascular treatment for thrombotic arterial occlusion of the CA or SMA in the setting of atherosclerotic disease near the vessel origin, and to identify possible prognostic factors in this group of patients.

## Material and Methods

### Study Population

Approval for this retrospective analysis was waived by the institutional review board (EK 241/20). The electronic medical records of our tertiary care medical center were searched to identify patients who underwent stent implantation to treat AMI from January 2011 to December 2019. All consecutive patients with AMI due to occlusion or subocclusion (> 90% reduction of vessel caliber) of the CA or SMA resulting from arterial thrombosis in the setting of atherosclerotic disease within the proximal 4 cm of the vessel, who were treated by means of PTA and stent implantation, were included. Patients who presented with AMI from other causes (i.e., arterial embolism, venous thrombosis, non-occlusive mesenteric ischemia) were excluded.

### Diagnostic and Therapeutic Algorithm

All patients with clinical signs and symptoms of AMI routinely received a contrast-enhanced CT for initial evaluation. CT scans were performed using a 128-slice CT scanner (SOMATOM Definition Flash, Siemens Healthcare) or a 40-slice CT scan (SOMATOM Definition AS, Siemens Healthcare). An iodinated contrast medium (Ultravist®-370, Bayer AG) in a dosage of 1.5 ml/kg was administered at a rate of 3.5–5 ml/sec in every patient. Image acquisition was performed in arterial and portal venous phase.

Patients with imaging findings consistent with acute mesenteric ischemia were immediately discussed in a multi-disciplinary team consisting of an interventional radiologist, a vascular surgeon and an abdominal surgeon.

**Fig. 1:**
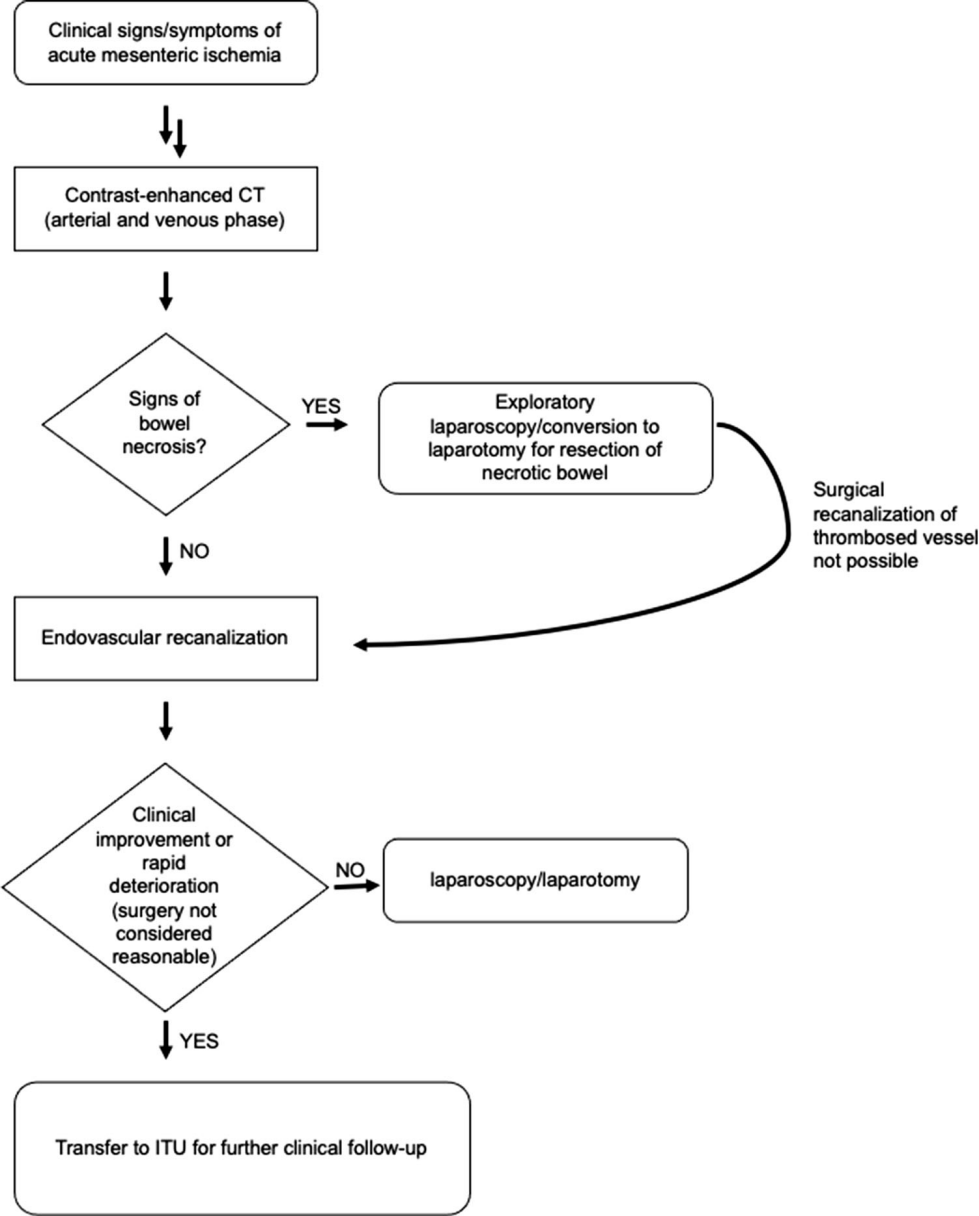
Flowchart of the diagnostic and therapeutic algorithm

Patients with an acute thrombotic occlusion/subocclusion of the CA or SMA and signs of bowel necrosis on CT [13] were transferred immediately to the operating theater for surgical resection of necrotic bowel parts. If no surgical bypass was feasible, these patients were re-evaluated for eventual endovascular recanalization after surgery. Patients with an acute thrombotic occlusion/subocclusion of the CA or SMA without signs of bowel necrosis (i.e., negative findings concerning bowels or signs of bowel ischemia) underwent endovascular recanalization [6, 14]. After recanalization, patients were discussed again in a multi-disciplinary team: if they showed hemodynamical and clinical improvement or were rapidly deteriorating so that no further invasive procedures appeared reasonable, they were transferred to the ITU for further monitoring. If they were hemodynamically unstable, complained of persistent abdominal pain or showed rising serum lactate levels and were considered good surgical candidates, they would undergo explorative laparoscopy and possibly bowel resection if necessary (after conversion to laparotomy). A flowchart illustrates the diagnostic and therapeutic algorithm for management of patients with AMI at our institution (Fig. 1).

### Endovascular Procedure

In case of an isolated occlusion of the SMA or CA, the respective occluded vessel was the only target vessel for recanalization and stent implantation. In case of occlusion of both vessels, recanalization of the SMA was the primary goal. If easily achievable (< 10 min for the passage of the occlusion), the CA was also recanalized and treated in order to improve blood flow via the anastomoses between the SMA and CA. Whenever recanalization of the SMA was not possible, for example due to rigid and/or long calcifications, the CA was recanalized as a “bail out” to restore blood flow to the intestines via the gastroduodenal artery and other anastomoses to the SMA. Technical success of the intervention was defined as the successful reestablishment of patency of the target vessel with blood flow into the peripheral side branches with a residual stenosis of < 30% after stent implantation.

**Fig. 2:**
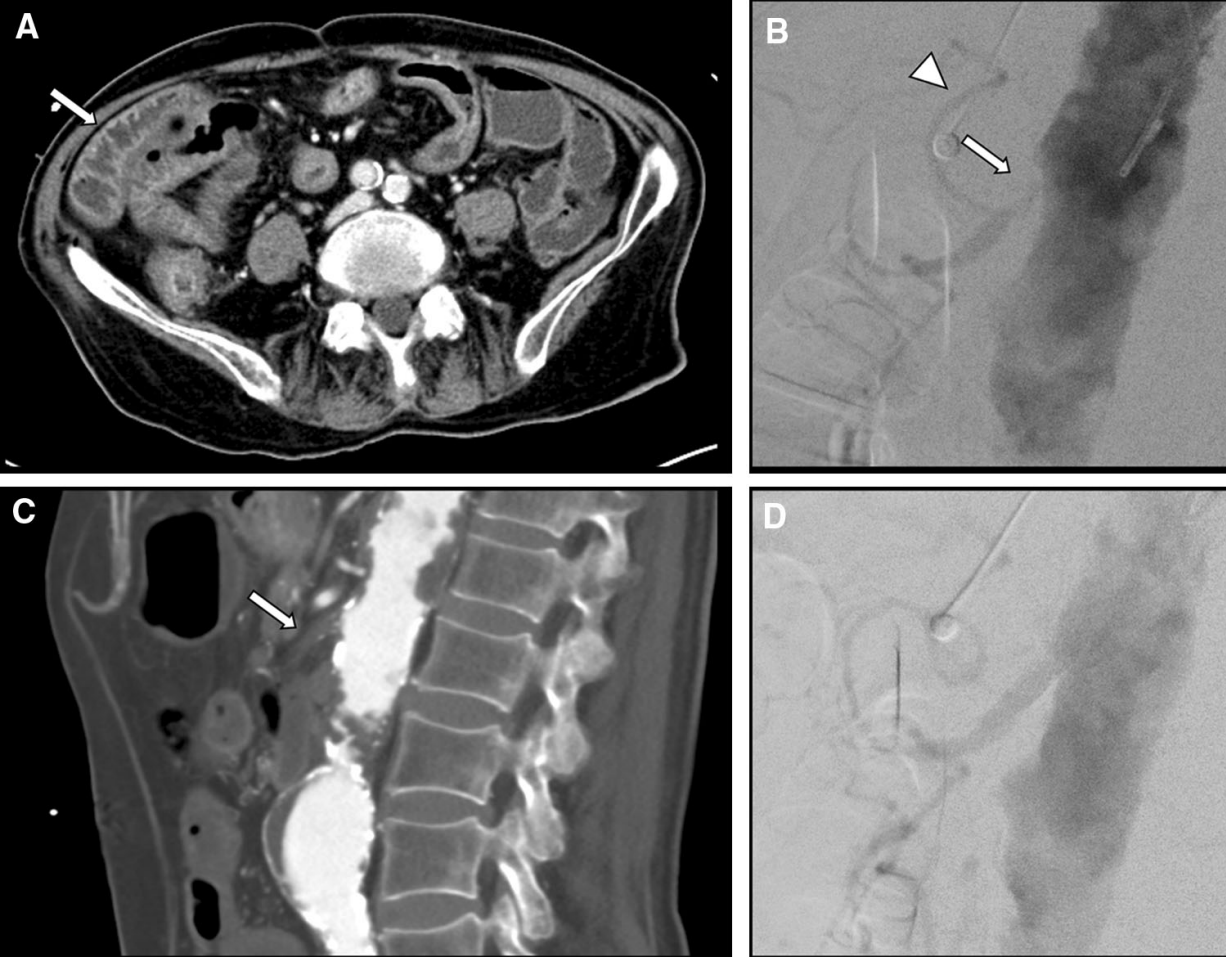
Sample case. 91-year-old male patient who presented to the emergency room because of acute worsening abdominal pain, as well as nausea and diarrhea. On the initial CT (**A)** edematous swelling of the bowel wall of the terminal ileum and right colon (arrow) was noted; the sagittal reconstruction of the arterial phase images showed a complete occlusion of the superior mesenteric artery at the origin resulting from arterial thrombosis due to severe atherosclerosis. The patient was immediately transferred to the angiography suite for emergency endovascular revascularization. Digital subtraction angiography confirmed the proximal occlusion of the superior mesenteric artery (**B**, arrow) with perfusion of the distal parts of the SMA and its branches via the gastroduodenal artery (arrowhead). Digital subtraction angiography after recanalization with stent implantation (6 × 16 mm Formula® 418 Vascular Balloon-Expandable Stent, Cook Medical, Bloomington) showed restored antegrade perfusion of the SMA (**D**)

The procedure was performed in 28/40 cases via a right transfemoral and in 12/40 cases via a left transbrachial approach. After introduction of a 6, 6.5 or 7F-sheath (Flexor® Check-Flo® Introducer or Flexor® Tuohy-Borst Side-Arm Introducer with Ansel modification, Cook Medical, Bloomington, USA; Super Arrow-Flex®, Teleflex Incorporated, Wayne, Pennsylvania, USA; Destino™ Twist, OSCOR Inc ©, Palm Harbor, Florida, USA), the stenosed or occluded segment of the vessel was crossed using a 0.035″ Guidewire (Radifocus® Guide Wire M, Terumo Corporation, Tokyo, Japan) or a 0.014″ microguidewire (Fathom™, Boston Scientific Corporation, Marlborough, USA) and a 2.4F Microcatheter (Progreat®, Terumo Corporation, Tokyo, Japan; DirexionTM, Boston Scientific Corporation, Marlborough, USA). After confirming the intraluminal position of the (micro)catheter distal to the stenosis with contrast medium injection, pre-dilation of the stenosis was usually performed using an undersized balloon catheter (3–4 mm diameter, Sterling™ Balloon Catheter, Boston Scientific Corporation, Marlborough, USA). In all patients, a balloon-expandable stent was deployed (Fig. 2) (Formula® 535 Vascular Balloon-Expandable Stent, Cook Medical; Omnilink Elite Vascular Balloon-Expandable Stent System, MULTI-LINK VISION RX Coronary Stent System, Herculink Elite®, Abbott Vascular). Data on duration of the intervention were noted for each procedure.

Anticoagulation with i.v. administration of heparin (1000 I.U./h) was performed for 24 h after the procedure; dual antiplatelet therapy with clopidogrel 75 mg/day for 6 weeks and aspirin 100 mg/day lifelong was recommended. All patients also received a loading dose of 225 mg clopidogrel immediately after the endovascular procedure, except for those patients who were transferred to the operating theater, as the clopidogrel loading dose was withheld until after surgery in these patients.

### Clinical Data Collection

Electronic medical records were reviewed regarding the initial presentation of symptoms (outpatient/hospitalized patient, ITU), type of surgical treatment (diagnostic laparoscopy vs. resection of necrotic bowel parts), history of smoking, history of symptoms of chronic mesenteric ischemia (e.g., abdominal angina) and the post-interventional survival. Additionally, elevated pre-interventional serum lactate levels, pre-interventional total leucocyte count (TLC) pre-interventional ratio between platelets and lymphocytes (PLR) as well as between neutrophils and lymphocytes (NLR) were collected. 30-day survival was calculated. If available, post-interventional CT or ultrasound images were evaluated regarding patency of stent during follow-up. Pre-interventional CT scans were retrospectively reviewed by two vascular and interventional radiologists, who were blinded to the outcome of the intervention and consensually evaluated the patency of inferior mesenteric artery (IMA). In addition, imaging signs of bowel necrosis (absence of wall enhancement, pneumatosis, free peritoneal gas) or ischemia (thickening of bowel wall, hyperattenuating bowel wall, mesenteric stranding, ascites) on the pre-interventional CT were recorded [13].

### Statistical Analysis

Quantitative measurements were expressed by median and interquartile range (IQR). Univariate binomial logistic regression analyses were used to determine the possible association between 30 day survival and the following parameters:

- Sex
- Age
- Occurrence of ischemia in an outpatient or inpatient setting
- Occurrence of ischemia during an ITU stay
- History of smoking
- History of chronic mesenteric ischemia
- Signs of bowel necrosis on pre-interventional CT
- One- or two-vessel disease (CA, SMA)
- Patency of the IMA
- Pre-interventional serum lactate levels
- Pre-interventional TLC
- Pre-interventional PLR
- Pre-interventional NLR

Statistical significance was considered to be present with a *p*-value ≤ 0.05. If more than one parameter had yielded *p*-values ≤ 0.1, a multivariable binomial regression analysis would have been performed. Kaplan–Meier method was used to calculate the median overall survival of patients who did not die in the first 30 days after the procedure. Statistical analysis was performed with SPSS 27 (IBM, Armonk, New York, USA).

## Results

### Patient Cohort

A total of 40 patients (18 males, 22 females) with a mean age of 74 years (IQR: 63–80 years) were included in this retrospective analysis. 17/40 patients (43%) presented as outpatients in our emergency department with acute abdominal pain and were diagnosed with acute mesenteric ischemia after emergency CT. The remaining 23/40 patients (57%) were inpatients who had been hospitalized due to various pre-existing conditions: Sepsis due to various infectious diseases (*n* = 8), postoperative situation after major abdominal (*n* = 5) or cardiovascular surgery (*n* = 3), or with gastric or duodenal ulcer with or without active bleeding (*n* = 3), ischemic or hemorrhagic stroke (*n* = 3), or mitral valve insufficiency (*n* = 1). 15/40 patients (37.5%) were hospitalized in an ITU when the AMI occurred, whereas the remaining 25/40 patients (62.5%) were either outpatients or hospitalized on a normal ward at the time when symptoms of AMI started. 14/40 patients (35%) had a history of smoking, and 9/40 patients (22.5%) reported that they had previously experienced symptoms of abdominal angina.

### Pre-Interventional Imaging

A total of 17 of the 40 patients (42.5%) had imaging evidence of bowel necrosis (2/17) or ischemia (15/17) on pre-interventional CT. 34/40 patients (85%) had a patent IMA on the pre-interventional CT.

### Blood Tests

Median TLC was 14.85/nl (IQR: 8.45–21.60/nl). Serum lactate levels were measured in 26/40 patients (65%) before the start of intervention and were elevated above normal levels in 21 out of 26 patients (80%). Median serum lactate level in these 26 patients was 73.0 mmol/L (IQR: 1.7–6.12 mmol/L). A white blood cell differential was performed in 16/40 patients. The median number of platelets was 190/nl (IQR: 159.75–207.5/nl), the median number of lymphocytes was 0,56/nl (IQR:0.30–0.94/nl), and the median number of neutrophils was 9.29/NL (IQR: 6.48–17.04/nl). The median PLR was 344 (IQR: 232–791), and the median NLR was 29 (IQR: 19–50).

### Technical Success of AMI Stenting

Revascularization of the target vessel was successful in 36/40 patients (90%). 11/40 patients (27.5%) had an isolated occlusion of the CA and 7/40 patients (17.5%) had an isolated occlusion of the SMA, all of which were successfully recanalized; therefore, 18/40 patients (45%) presented one-vessel disease. 22/40 patients (55%) had AMI with occlusions of both SMA and CA (two-vessel disease); in 7/22 patients (32%) a recanalization of both CA and SMA was possible, in 11/22 (50%) patients the only SMA was recanalized, in 4/22 patients (18%) the CA was recanalized as a “bail-out” since recanalization of the SMA was not possible and showed a reperfusion of the SMA distal to the gastroduodenal arcade. The median duration of the procedures, once vascular access had been established, was 87 min (IQR: 73–103 min). No peri-interventional complications (distal embolization, dissection) occurred. Follow-up with CT or Doppler ultrasound was available in only 14/40 patients with a median of 7.5 days after the intervention (IQR: 1–24 days), showing patency of the stent in 12/14 cases (86%).

### Additional Surgical Treatment Before/After Revascularization

The 2/40 patients (5%) who showed signs of extensive bowel necrosis on CT underwent resection of small bowel and colon before proceeding to the angiography suite for endovascular recanalization. 25 out of 40 patients (62.5%) underwent abdominal laparoscopy or laparotomy after revascularization, and in 12/25 patients (48%) necrotic parts of the bowel were resected, while in the remaining 13/25 patients (52%), no additional surgical measures were needed or possible. The remaining 13/40 patients (32.5%) did not undergo surgery after revascularization.

### Patient Outcome

25 out of 40 patients died within the first 30 days, thus the 30-day-mortality rate was 62.5%. 10/25 patients died because of septic shock and 15/25 patients eventually died because of cardiogenic shock. In patients who died within the first 30 days, follow-up was available in 8 cases (median 1 day, IQR: 1; 2 days) showing a stent patency rate of 6/8 (75%).

Age (*p* = 0.540), sex (*p* = 0.804) of patients, history of smoke (*p* = 0.608) or chronic mesenteric ischemia (*p* = 0.770), onset of symptoms by outpatient or inpatient (*p* = 0.680), in ITU or not in ITU (*p* = 0.800), one- vs. two-vessel disease(*p* = 0.935), signs of bowel necrosis or ischemia (*p* = 0.395) or patency of IMA on pre-interventional CT (*p* = 0.306) were not statistically significantly associated with 30 day survival. Likewise, NLR (*p* = 0.319), PLR (*p* = 0.233), TLC (*p* = 0.175) and pre-interventional elevation of serum lactate (*p* = 0.244) showed no statistically significative association with 30-day survival. Further details of all analyzed data are shown in Table 1.

**Table 1:** Features of patients and results of regression analyses of possible predictors for 30-day mortality

	Survival < 30 days (25 patients)	Survival > 30 days (15 patients)	Hazard ratio (95% confidence interval)	*p* value
Sex (M/F)	11/14	6/9	1.179 (0.321; 4.326)	0.804
Age	74 (62; 80)	70 (64; 79)	1.202 (0.958; 1.086)	0.540
Outpatient/inpatient occurrence of AMI	10/15	7/8	1.312 (0.361; 4.777)	0.680
Not ITU/ITU occurrence of AMI	16/9	9/6	0.844 (0.226; 3.148)	0.800
Smoke	32% (8/25)	40% (6/15)	0.706 (0.186; 2.673)	0.608
Chronic mesenteric ischemia	24% (6/25)	20% (3/15)	1.263 (0.265; 6.029)	0.770
Signs of bowel ischemia or necrosis on pre-interventional CT	48% (12/25)	33% (5/15)	1.800 (0.464; 6.976)	0.395
One-/two-vessel disease (CA + SMA)	11/14	7/8	1.114 (0.308; 4.028)	0.870
Patency of IMA	80% (20/25)	93% (14/15)	3.250 (0.340; 31,074)	0.306
Pre-interventional elevation of serum lactate	50% (9/18)	86% (6/7)	0.333 (0.053; 2.115)	0.244
Pre-interventional neutrophil/lymphocyte ratio (NLR)	20 (20; 50)	42 (33; 44)	0.987 (0.961; 1.013)	0.319
Pre-interventional platelet/lymphocyte ratio (PLR)	273 (245; 516)	763 (715; 942)	0.999 (0.998; 1.001)	0.233
Pre-interventional total leucocyte count (TLC)	17.4 (8.2; 23.2)	11.5 (9.2; 18.2)	1.064 (0.973; 1.163)	0.175

**Fig. 3:**
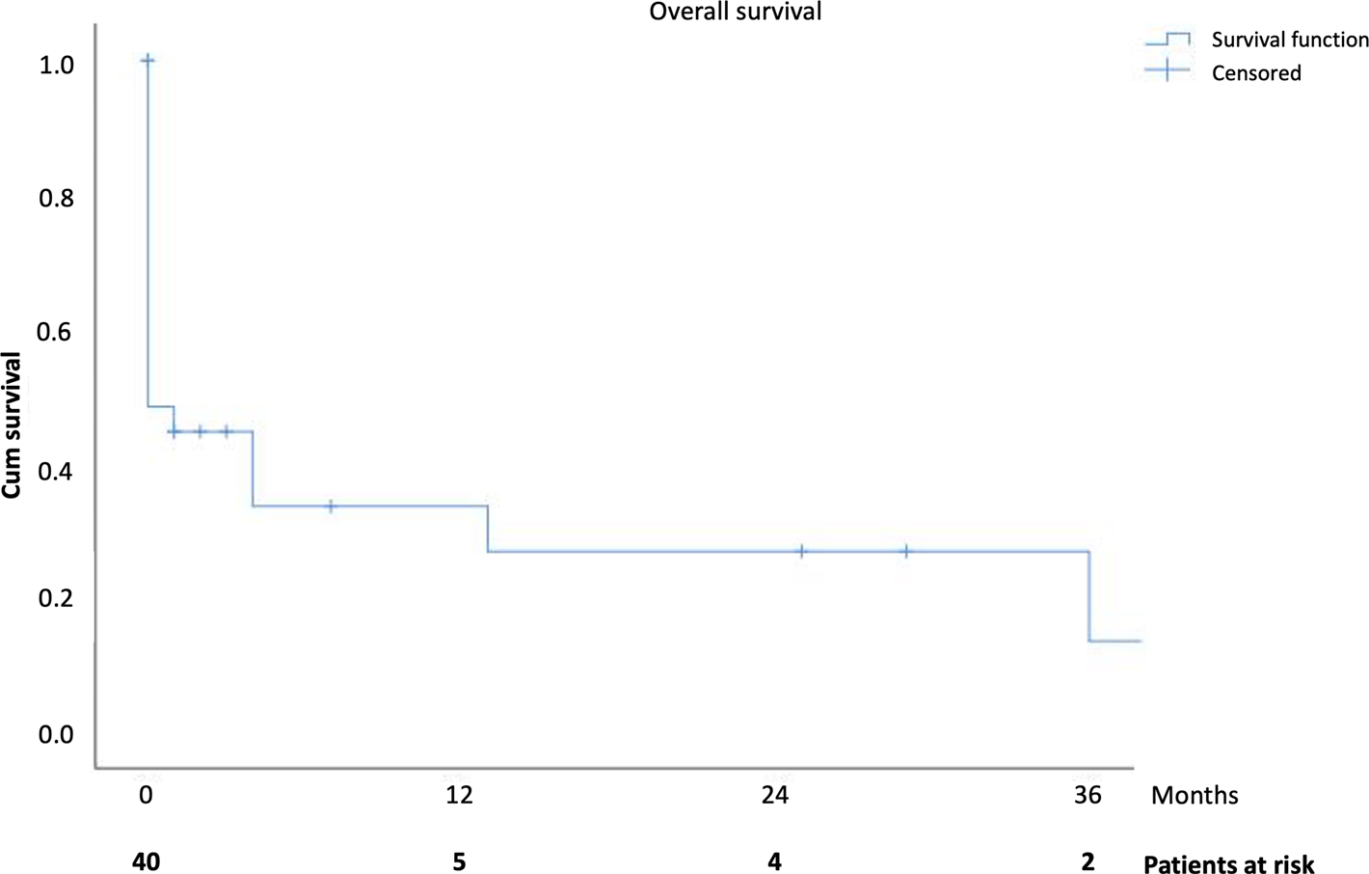
Kaplan–Meier mortality curve

Median overall survival in patients who did not die in the first 30 days (*n* = 15) was 36 ± 18 months. Kaplan–Meier curve is shown in Fig. 3.

## Discussion

In this retrospective analysis, only about one-third of patients with AMI from “acute-on-chronic” arterial thrombosis of SMA and/or CA survived the first post-interventional month despite technically successful revascularization in 90% of patients and “bail-out”-revascularization of the CA in the remaining 10% of patients. Clinical history, imaging, and laboratory data all proved to be unreliable to predict the patient outcome.

Among the different causes of AMI, acute thrombosis of the ostia of the CA or SMA has been described to be associated with the worst prognosis, with in-hospital mortality rates up to 87% [15]. The reason for the particularly poor outcome in patients with acute arterial mesenteric thrombosis is that it usually affects patients older than 70 years suffering from atherosclerosis with long-standing stenoses at the origins of the SMA or CA [13], which often results in greater bowel involvement in the acute setting compared with arterial embolization, which is usually more distally located [16]. However, data regarding mortality of patients with AMI vary widely in the literature. Block TA et al. reported on data of the Swedish Vascular Registry, including 21 open and 42 endovascular revascularizations of thrombotic and embolic occlusions of the SMA; patients treated for acute thrombosis either surgically or endovascularly showed a 30-day-mortality rate of 40% [17]. Likewise, the majority of studies on AMI are inhomogeneous regarding patient selection and include patients with AMI deriving from different causes [18], although the underlying pathophysiology and treatment varies significantly between those different causes [6]. Therefore, these different subgroups of patients with AMI should be analyzed separately when investigating patient outcome.

Endovascular recanalization is the treatment of choice in patients with thrombotic arterial AMI without signs of intestinal necrosis [6]. Although prospective trials comparing endovascular and surgical treatments are lacking in the current literature, several meta-analyses [19–21] suggest that endovascular recanalization is associated with a lower mortality, complication rate and shorter length of hospitalization compared to open surgery [22, 23]. In our cohort, the rate of patients who received bowel resection after revascularization was 30% (12/40). Considering the poor prognosis of patients with AMI and since time plays a crucial role in prevention of bowel necrosis and survival [24], it would be useful to identify factors able to predict the outcome of patients after revascularization in order to improve management. If such factors were available, patients predicted to have a poor outcome could be triaged to undergo alternative treatment approaches such as open surgery or even hybrid retrograde open mesenteric stenting. The latter requires a dedicated infrastructure but has shown promising results in a previous study [25].

Several studies have suggested potential predicting factors for the outcome of patients with AMI. For example, the presence of elevated levels of serum lactate, a product of anaerobic glycolysis, has been described in several studies to be associated with presence of irreversible transmural necrosis [8, 9] and worse prognosis [11, 26]. Grotelüschen et al. showed that patients with AMI diagnosed during a hospital stay in an intensive care unit was associated with a worse prognosis in a cohort of 302 patients who underwent surgery due to AMI [11]. Augène et al. demonstrated that patients with higher PLR but not NLR value had significantly higher rate of mortality in a cohort of 106 patients with AMI caused by arterial embolism and thrombosis [10]. Age and delayed interventions were also described as prognostic factors in a cohort of 74 patients who mostly underwent surgical treatment [12]. Unfortunately, none of the potential predictors analyzed in the present study showed a statistically significant association with 30-day mortality.

It is widely accepted that time is critical in the treatment of AMI and therefore restoration of blood flow to the bowel is the main priority in these patients to avoid onset of irreversible necrosis of the bowel wall. Nevertheless, results of delayed diagnosis or treatment in predicting mortality after AMI are controversial in the literature [12, 17], and current data suggests that ischemic changes are reversible in the first six hours, although, as opposed to ischemic stroke, current guidelines do not provide precise information on proper timing limits for revascularization. Data on the time interval between onset of symptoms and treatment were unfortunately not available on our cohort, and this constitutes a major limitation of the study. However, we investigated whether the outcome of outpatients differs from the outcome of inpatients as a surrogate maker, since diagnosis and treatment should be faster in patients who are already hospitalized and found no statistically significant difference between these two groups. There are several other limitations to this study, such as its retrospective nature and limited number of patients which in turn limits the statistical power, especially regarding the analysis of factors that would determine patients’ outcome. However, we focused on patients with AMI deriving from occlusion of visceral arteries due to arterial thrombosis and excluded patients with all other sources of AMI. This limited the number of patients in our cohort, but was necessary, since different causes of AMI should be treated differently according to the current guidelines of the European Society for Trauma and Emergency Surgery (ESTES). Furthermore, the patient cohort is heterogenous due to the fact that these patients were treated under emergency conditions by different interventionalists and there was no standardized follow-up. However, this cohort comprises real-life data obtained at a tertiary care hospital, at which several patients are referred also from external institutions and may be transferred back to these institutions once emergency treatment has been concluded. Furthermore, we did not grade ischemic changes of the bowel on the pre-interventional CT and instead only searched for evidence of irreversible bowel necrosis. Lastly, most patients with AMI have additional underlying medical conditions such as coronary artery disease and cerebrovascular disease, and those patients who were already hospitalized when the AMI occurred obviously had additional acute medical conditions as well. The post-interventional mortality may therefore be biased by these additional medical conditions.

In conclusion, the 30-day mortality rate remains undeniably high despite emergency endovascular stenting of the SMA and/or CA in patients with AMI due to acute arterial thrombosis. Due to the lack of prognostic factors for the clinical outcome in this patient cohort, which could possibly be used to able to guide patient management towards alternative treatments, we nevertheless encourage an immediate endovascular revascularization effort in all patients.
